# Retraction: A Retrospective Cohort Study of the Association Between Calcium Serum Level and Hypertension in Older Adults

**DOI:** 10.7759/cureus.r180

**Published:** 2025-04-30

**Authors:** Zia Sabah, Ahmed S Al Zomia, Mosab Deajim, Abdulrahman Alshahrani, Abdullah H Alamri, Ali M Alqahtani, Lama A Lahiq, Nasser A Alwaqdi, Berin Raj

**Affiliations:** 1 Department of Cardiology, King Khalid University, Abha, SAU; 2 College of Medicine, King Khalid University, Abha, SAU; 3 Department of Public Health, Prince Faisal Bin Khalid Cardiac Center, Abha, SAU; 4 Department of Public Health, University of Sunderland, Sunderland, GBR

The editors-in-chief have retracted this article due to multiple concerns regarding the integrity and validity of the reported data. Specifically, the study reports physiologically implausible mean serum calcium levels, raising serious questions about data accuracy. Additionally, discrepancies in the stated sample size, lack of appropriate control for confounding variables, over-interpretation of weak statistical correlations, and inconsistent reporting throughout the manuscript undermine the reliability of the findings. As a result, the conclusions drawn cannot be considered scientifically sound.

